# Attribution of net carbon change by disturbance type across forest lands of the conterminous United States

**DOI:** 10.1186/s13021-016-0066-5

**Published:** 2016-11-14

**Authors:** N. L. Harris, S. C. Hagen, S. S. Saatchi, T. R. H. Pearson, C. W. Woodall, G. M. Domke, B. H. Braswell, B. F. Walters, S. Brown, W. Salas, A. Fore, Y. Yu

**Affiliations:** 1 Ecosystem Services Unit, Winrock International, 2121 Crystal Drive Suite 500, Arlington, VA 22202 USA; 2 Applied Geosolutions, 55 Main Street Suite 125, Newmarket, NH 03857 USA; 3 NASA Jet Propulsion Laboratory, California Institute of Technology, Pasadena, CA 91109 USA; 4 USDA Forest Service, Northern Research Station, Saint Paul, MN 55108 USA; 5 Forests Program, World Resources Institute, 10 G Street NE Suite 800, Washington, DC 20002 USA

**Keywords:** Forests, Disturbance, Harvest, Insects, Fire, Drought, Greenhouse gas, Land use, Climate change, FIA, UNFCCC

## Abstract

**Background:**

Locating terrestrial sources and sinks of carbon (C) will be critical to developing strategies that contribute to the climate change mitigation goals of the Paris Agreement. Here we present spatially resolved estimates of net C change across United States (US) forest lands between 2006 and 2010 and attribute them to natural and anthropogenic processes.

**Results:**

Forests in the conterminous US sequestered −460 ± 48 Tg C year^−1^, while C losses from disturbance averaged 191 ± 10 Tg C year^−1^. Combining estimates of net C losses and gains results in net carbon change of −269 ± 49 Tg C year^−1^. New forests gained −8 ± 1 Tg C year^−1^, while deforestation resulted in losses of 6 ± 1 Tg C year^−1^. Forest land remaining forest land lost 185 ± 10 Tg C year^−1^ to various disturbances; these losses were compensated by net carbon gains of −452 ± 48 Tg C year^−1^. C loss in the southern US was highest (105 ± 6 Tg C year^−1^) with the highest fractional contributions from harvest (92%) and wind (5%). C loss in the western US (44 ± 3 Tg C year^−1^) was due predominantly to harvest (66%), fire (15%), and insect damage (13%). The northern US had the lowest C loss (41 ± 2 Tg C year^−1^) with the most significant proportional contributions from harvest (86%), insect damage (9%), and conversion (3%). Taken together, these disturbances reduced the estimated potential C sink of US forests by 42%.

**Conclusion:**

The framework presented here allows for the integration of ground and space observations to more fully inform US forest C policy and monitoring efforts.

## Background

The 2015 Paris Climate Change Agreement, with consensus from 192 signatories, calls for achieving a balance between anthropogenic emissions by sources and removals by sinks in the second half of this century [1]. Forests are currently responsible for the capture and storage of an estimated 25% of global anthropogenic emissions [2]. If Paris goals are to be achieved, further enhancement of forest-based carbon (C) removals to mitigate emissions in other sectors will be a critical component of any collective global strategy [3], especially as no alternative sink technologies have yet been proven at scale. Thus, spatially identifying terrestrial sources and sinks of carbon, and understanding them well enough to predict how they will respond to management decisions or future climate change, will pose major science and policy challenges in the years to come.

Remote sensing products can provide regular and consistent observations of Earth’s surface to help identify the condition of forest ecosystems and changes within them at a range of spatial and temporal scales [4]. Over the past several years, the remote sensing research community has used these products to monitor tropical deforestation, forest C stocks and associated C emissions, largely in support of REDD+ initiatives in developing countries [5–12]. In many developed countries, periodic national forest inventories form the basis of annual greenhouse gas (GHG) reporting to the United Nations Framework Convention on Climate Change (UNFCCC). The sample-based design of these inventories may offer little in the way of detailed and spatially-explicit information on the distribution of forest biomass [13], timing and location of timber harvesting in managed forests, or the cause and timing of other types of forest disturbances. If the ultimate aim of the Paris Agreement is to introduce practices that lead to reduced emissions and enhanced removals of C from the world’s managed forests, including in temperate and boreal biomes, then a lack of disaggregated, spatially-explicit information could pose challenges over the coming years related to knowledge of where changes are occurring and where interventions are likely to be most effective.

Several C budget models have been developed to simulate ecosystem response to climate drivers and other disturbances, and these models represent an established approach to estimating C fluxes at national to regional scales. For example, Canada’s National Forest Carbon Monitoring Accounting and Reporting System (NFCMARS) uses the Carbon Budget Model of the Canadian Forest Sector (CBM-CFS3), and is used also as a decision support tool for forest managers to quantify forest C dynamics at a landscape scale. Different models emphasize different aspects of ecosystem dynamics, with some accounting for competition between plant functional types, nutrient limitation, and natural disturbances. Time series of anthropogenic land-cover changes are usually prescribed based on spatially explicit data. The models can reflect spatial and temporal variability in C density and response to environmental conditions, but their modeled C stocks may differ markedly from observations [14].

Such models are not used explicitly in the GHG inventory for the US to report forest C fluxes. Instead, the current US inventory system uses the C stock-difference accounting approach [15] enabled by the annual national forest inventory conducted by the United States Department of Agriculture (USDA) Forest Service Forest Inventory and Analysis (FIA) program. The difference in C stocks in five C pools is estimated via sequential re-measurements of permanent ground inventory plots. When forest stocks decline, it is assumed that C emissions have occurred from the land to the atmosphere if not reconciled with a transfer to another land use category. Conversely, when forest C stocks increase it is assumed that C has been sequestered from the atmosphere by terrestrial vegetation. In this way, estimated net C change in the US forest sector is the integrated result of both anthropogenic and natural processes—harvest, land use change, fire, drought, insect infestation, wind damage—all of which influence the magnitude of forest C stocks in each pool. Results are most statistically robust when compiled at large spatial scales (e.g., state or regional), such that quantification of finer-scale spatial patterns is less precise. Though changes are well constrained via sequential re-measurements on inventory plots, the US [16, 17] has only recently begun using methods to disaggregate the effects of various disturbance types on forest stocks and fluxes (although this separation is not a requirement of IPCC Good Practice Guidance, [18]).

The objective of this study was to synthesize information from remote sensing observations of forest carbon stocks and disturbance with information collected by various US agencies into a framework that (1) more explicitly attributes C losses to major disturbance types (land use change, harvesting, forest fires, insect damage, wind damage and drought); and (2) disaggregates net C change into relevant IPCC reporting categories of non-forest land converted to forest land, forest land converted to non-forest land, and forest land remaining forest land. This framework allows for the integration of ground and space observations to more fully inform US forest C policy and monitoring efforts.

## Methods

**Fig. 1:**
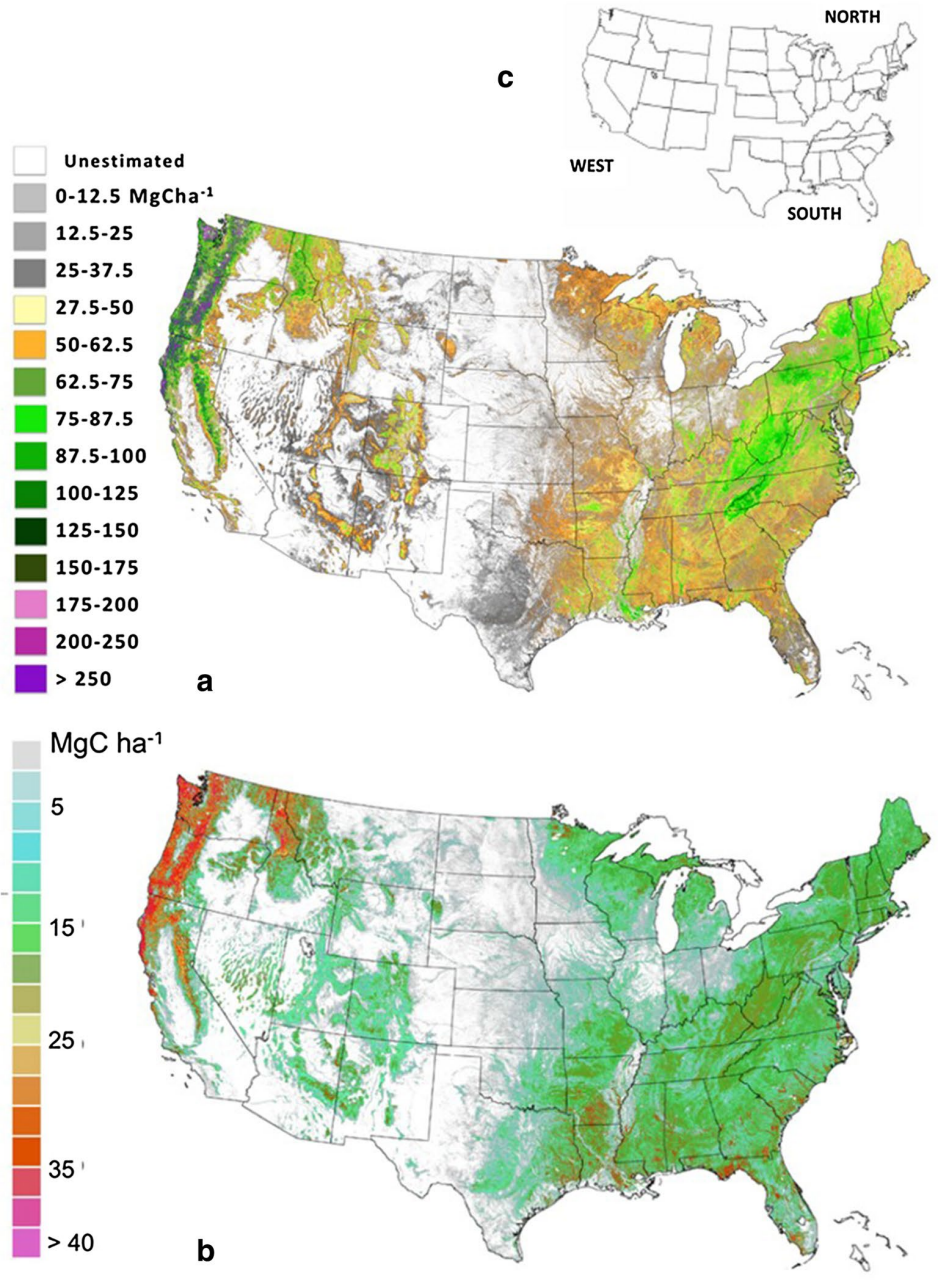
**a** Map of aboveground live woody biomass carbon density (Mg C ha^−1^) and **b** uncertainty across forest lands of the conterminous US at 1-ha resolution for circa the year 2005. **c** The regional analysis was performed by dividing the US into three sub-regions as recommended by Heath and Birdsey [19]. The above and belowground carbon density maps and the uncertainty maps can be downloaded from NASA’s distributed Data Active Archive Center (http://dx.doi.org/10.3334/ORNLDAAC/1313)

We built a spatially-explicit empirical model that combines information from many data sources to infer disturbance and resulting C dynamics within each hectare of forest land in the 48 conterminous states of the US, totaling an area of more than 2.1 million km^2^. For the purposes of regional comparison and analyses, we divided the US into three broad regions (North, South, West) based on similar histories of forestland use ([19], Fig. 1) and into nine smaller subregions based on those used in the US FIA program. Forest types were defined as hardwood or softwood, following the National Land Cover Data (NLCD) classification (deciduous forest class: hardwoods; evergreen forest class: softwoods). The time period of analysis is 1 January 2006 to 31 December 2010.

### Data inputs

#### Forest area map (2005)

Forest extent in the base year 2005 was determined from the NLCD and the global tree cover and tree cover change products of Hansen et al. [8]. Specifically, an area was determined to be forested if categorized as hardwood or softwood in the NLCD 2006 dataset^1^ and, according to the Hansen et al. [8] dataset, it (a) met the tree cover threshold of 25% in the year 2000 and was not lost between 2001 and 2005 or (b) did not meet the tree cover threshold of 25% in 2000 but was identified as having gained tree cover (i.e., afforestation/reforestation) between 2000 and 2012. The NLCD has been shown to significantly underestimate tree cover [20] and thus the forest area estimates used in this analysis—defined by both NLCD and Hansen et al. [8]—are likely to be conservative. However, these two data products currently represent the best available spatially explicit data for forest extent in the conterminous US (CONUS).

#### Forest biomass density maps (circa 2005)

We developed maps of C stocks (50% of biomass) in aboveground live biomass in US forest land as part of NASA’s C Monitoring System (CMS) program based on a combination of remote sensing observations and FIA data (Fig. 1). The overall methodology used in mapping the aboveground live forest biomass C density is described in Saatchi et al. [5]. After filtering for cloud effects, slopes, and signal-to-noise ratio, more than 700,000 samples of lidar (light detecting and ranging) data acquired between 2003 and 2008 from the Geoscience Laser Altimeter System (GLAS), onboard the Ice, Cloud and land Elevation Satellite (ICESat) were used as samples of the vertical structure of US forest land. We used the Lorey’s height [21] measured in 65,000 single-condition FIA plots (i.e., plots with a single domain mapped on each plot) to calibrate the lidar-derived height metric and used the relationship between Lorey’s height and aboveground C density for 28 forest types to convert the lidar data into estimates of aboveground live C density. All FIA plots with a probability of disturbance causing reduced canopy cover (<50%) were removed from the height-biomass model development to reduce any potential discrepancy between ground and lidar height metrics. Lidar-derived biomass samples were then extrapolated over the landscape using a combination of optical and radar satellite imagery that captures the variations of forest structure and cover to create wall-to-wall maps of forest aboveground live biomass C density. We used nine remote sensing imagery layers as spatial predictor variables. Optical and thermal data from Landsat imagery (bands 3, 4, 5 and 7) were aggregated to 100 m spatial resolution from 30 m native resolution along with the leaf area index derived from Landsat imagery [22]. In addition, we used the advanced land observing satellite (ALOS) phased area L-band synthetic aperture radar (PALSAR) imagery at two polarizations (HH and HV backscatter) along with topographical data of surface elevation and slope from Shuttle Radar Topography Mission (SRTM) resampled to 100 m resolution from 20 and 30 m native resolutions, respectively. ALOS PALSAR plays an important role in quantifying variation in forest biomass. In particular, the HV polarization provides the largest contribution among the data layers to predicted biomass because it has a strong direct sensitivity to biomass up to 100–150 Mgha^−1^ (depending on forest type), is less impacted by soil moisture and other environmental variables, and may contribute significantly in extrapolating larger biomass forests through texture and spatial correlation. Similarly, SRTM data include information on topography and also forest height. We used the national elevation data (NED) to represent the ground surface elevation and used the difference between SRTM and NED as an indicator of forest height. This variable also contributed significantly to explaining the spatial variation of biomass over forests with biomass values >150 Mgha^−1^.

The aboveground C density samples derived from GLAS data were combined with satellite imagery using the maximum entropy estimation (MaxEnt) algorithm to estimate aboveground biomass density for each 1-ha pixel. MaxEnt is a probability-based algorithm that estimates the posterior likelihood distribution of a variable by maximizing the entropy of said probability distribution while maintaining the constraints provided by the training samples [23]. We selected a random subset consisting of 70% of the samples (~500,000 samples) for model input and used the remaining 30% for model evaluation and validation. The product from the MaxEnt estimator includes both the mean aboveground carbon (AGC) density for each 1-ha pixel and the estimation of the error derived from a Bayesian probability estimator for each pixel. Spatial uncertainty analysis and uncertainty propagation were used to evaluate the overall uncertainty of AGC at the pixel level. This process included the quantification of error at each step of the process and the use of the Gaussian error propagation approach:

$${\text{Error}} = \sqrt {\varepsilon_{{{\mathbf{measurement}}}}^{{\mathbf{2}}} + \varepsilon_{{{\mathbf{allometry}}}}^{{\mathbf{2}}} + \varepsilon_{{{\mathbf{sampling}}}}^{{\mathbf{2}}} + \varepsilon_{{{\mathbf{prediction}}}}^{{\mathbf{2}}} }$$

where each of the terms are the relative errors at that pixel and represent the measurement errors of lidar for capturing the forest height, the error associated with the lidar aboveground C allometry model for each forest type, the error associated with sampling the 1-ha pixel with GLAS footprint size (~0.25 ha), and the MaxEnt prediction error. In evaluating the errors at the state and county level, we also included the spatial correlation of the prediction error from the MaxEnt approach [24].

In the FIA, belowground forest biomass is quantified using a root-shoot ratio [25]. Knowledge of root biomass dynamics is fundamental to improving our understanding of carbon allocation and storage in terrestrial ecosystems [26]. We used the relationship between belowground carbon (BGC) and AGC from the FIA data to develop a BGC spatial distribution at the same scale as AGC [5, 27]. In estimating the uncertainty in BGC, we followed the same approach as AGC with the addition of including the errors associated with the model used in relating AGC to BGC.

#### FIA stock change data (2006–2010)

To estimate average net changes in the stock of live AGC and BGC between 2006 and 2010 in forests disaggregated by disturbance type, we queried the FIA database (http://apps.fs.fed.us/fiadb-downloads/datamart.html) to extract more than 141,000 records associated with re-measured permanent plots, where each extracted record represents a “condition” (i.e., domain(s) mapped on each plot according to attributes such as land use, forest type, stand size, ownership, tree density, stand origin, and/or disturbance history) of a measured plot at two points in time, typically 5 years apart. Disturbed plots were stratified into a lookup table by geographic region (North, South, or West), forest type (hardwood or softwood), disturbance type (fire, insect, wind, conversion, or harvest), and disturbance intensity (Table 1). A similar lookup table was developed for undisturbed plots stratified by geographic region, forest type, and base C stock in the year 2005 (Table 2).

**Table 1:** Look-up table of annual fractional change (average = µ; standard error = σ) in aboveground carbon (AGC) and belowground carbon (BGC) in disturbed forests based on FIA plot data

Region	Forest type	Disturbance	Initial C	N	AGC µ	AGC σ	BGC µ	BGC σ
North	Softwood	Fire	Low	2	−0.003	0.012	−0.001	0.013
North	Softwood	Fire	Medium	3	−0.052	0.031	−0.053	0.031
North	Softwood	Fire	High	5	−0.150	0.030	−0.157	0.030
North	Softwood	Weather	Low	63	−0.013	0.016	−0.014	0.016
North	Softwood	Weather	High	10	−0.163	0.013	−0.169	0.013
North	Softwood	Insect	Low	85	−0.003	0.007	−0.003	0.008
North	Softwood	Insect	Medium	82	−0.044	0.023	−0.046	0.023
North	Softwood	Insect	High	45	−0.126	0.035	−0.133	0.032
North	Softwood	Harvested	Low	521	−0.046	0.035	−0.048	0.036
North	Softwood	Harvested	High	246	−0.152	0.026	−0.158	0.025
North	Hardwood	Fire	Low	40	−0.003	0.009	−0.003	0.009
North	Hardwood	Fire	Medium	29	−0.045	0.024	−0.048	0.023
North	Hardwood	Fire	High	11	−0.131	0.034	−0.136	0.034
North	Hardwood	Weather	Low	412	−0.011	0.016	−0.011	0.016
North	Hardwood	Weather	High	34	−0.160	0.017	−0.164	0.016
North	Hardwood	Insect	Low	656	−0.002	0.008	−0.002	0.008
North	Hardwood	Insect	Medium	432	−0.045	0.020	−0.046	0.020
North	Hardwood	Insect	High	118	−0.132	0.029	−0.136	0.028
North	Hardwood	Harvested	Low	2177	−0.047	0.035	−0.047	0.035
North	Hardwood	Harvested	High	806	−0.154	0.023	−0.157	0.023
South	Softwood	Fire	Low	127	−0.002	0.007	−0.003	0.008
South	Softwood	Fire	Medium	174	−0.048	0.021	−0.052	0.022
South	Softwood	Fire	High	52	−0.124	0.027	−0.131	0.028
South	Softwood	Weather	Low	78	−0.016	0.016	−0.017	0.016
South	Softwood	Weather	High	16	−0.161	0.026	−0.168	0.023
South	Softwood	Insect	Low	46	−0.002	0.008	−0.004	0.008
South	Softwood	Insect	Medium	66	−0.054	0.022	−0.059	0.023
South	Softwood	Insect	High	60	−0.135	0.030	−0.142	0.029
South	Softwood	Harvested	Low	1787	−0.044	0.034	−0.048	0.036
South	Softwood	Harvested	High	586	−0.149	0.025	−0.157	0.024
South	Hardwood	Fire	low	112	−0.002	0.008	−0.003	0.008
South	Hardwood	Fire	Medium	86	−0.042	0.021	−0.045	0.022
South	Hardwood	Fire	High	37	−0.131	0.033	−0.139	0.030
South	Hardwood	Weather	Low	484	−0.014	0.016	−0.015	0.016
South	Hardwood	Weather	High	32	−0.162	0.019	−0.167	0.017
South	Hardwood	Insect	Low	145	0.000	0.013	−0.002	0.011
South	Hardwood	Insect	Medium	121	−0.047	0.022	−0.051	0.022
South	Hardwood	Insect	High	38	−0.133	0.031	−0.138	0.031
South	Hardwood	Harvested	Low	1235	−0.048	0.036	−0.051	0.036
South	Hardwood	Harvested	High	609	−0.146	0.029	−0.152	0.027
West	Softwood	Fire	Low	13	−0.007	0.008	−0.007	0.008
West	Softwood	Fire	Medium	8	−0.049	0.023	−0.050	0.026
West	Softwood	Fire	High	0	−*0.126*	*NA*	−*0.133*	*NA*
West	Softwood	Weather	Low	5	−0.003	0.008	−0.003	0.008
West	Softwood	Weather	High	0	−*0.162*	*NA*	−*0.168*	*NA*
West	Softwood	Insect	Low	12	0.001	0.007	0.001	0.007
West	Softwood	Insect	Medium	3	−0.041	0.016	−0.044	0.018
West	Softwood	Insect	High	0	−*0.131*	*NA*	−*0.138*	*NA*
West	Softwood	Harvested	Low	28	−0.027	0.030	−0.028	0.031
West	Softwood	Harvested	High	0	−*0.150*	*NA*	−*0.157*	*NA*
West	Hardwood	Fire	Low	4	−0.002	0.008	−0.002	0.008
West	Hardwood	Fire	Medium	3	−0.057	0.021	−0.059	0.021
West	Hardwood	Fire	High	0	−*0.131*	*NA*	−*0.138*	*NA*
West	Hardwood	Weather	Low	0	−*0.013*	*NA*	−*0.013*	*NA*
West	Hardwood	Weather	High	0	−*0.161*	*NA*	−*0.165*	*NA*
West	Hardwood	Insect	Low	13	−0.003	0.008	−0.003	0.009
West	Hardwood	Insect	Medium	3	−0.041	0.025	−0.044	0.028
West	Hardwood	Insect	High	0	−*0.132*	*NA*	−*0.136*	*NA*
West	Hardwood	Harvested	Low	4	−0.039	0.031	−0.039	0.033
West	Hardwood	Harvested	High	0	−*0.151*	*NA*	−*0.155*	*NA*

*Italics* imputed from other regions

**Table 2:** Look-up table of annual fractional change (average = µ; standard error = σ) in aboveground carbon (AGC) and belowground carbon (BGC) in undisturbed forests, based on FIA plot data

Region	Forest type	Drought	Initial C	n	AGC µ	AGC σ	BGC µ	BGC σ
North	Softwood	No	<25	5167	0.064	0.135	0.080	0.199
North	Softwood	No	25–50	3459	0.023	0.034	0.023	0.034
North	Softwood	No	50–100	2085	0.016	0.024	0.016	0.024
North	Softwood	No	≥100	345	0.013	0.034	0.013	0.034
North	Softwood	Yes	<25	50	0.028	0.030	0.031	0.035
North	Softwood	Yes	25–50	50	0.008	0.034	0.008	0.035
North	Softwood	Yes	50–100	12	0.016	0.040	0.016	0.040
North	Softwood	Yes	≥100	2	0.013	0.017	0.013	0.016
North	Hardwood	No	<25	12,559	0.074	0.102	0.087	0.131
North	Hardwood	No	25–50	13,656	0.025	0.036	0.025	0.036
North	Hardwood	No	50–100	14,173	0.014	0.026	0.014	0.026
North	Hardwood	No	≥100	3265	0.010	0.030	0.010	0.030
North	Hardwood	Yes	<25	19	0.016	0.058	0.016	0.062
North	Hardwood	Yes	25–50	12	0.006	0.040	0.006	0.041
North	Hardwood	Yes	50–100	7	0.001	0.026	0.000	0.027
North	Hardwood	Yes	≥100	1	0.006	NA	0.005	NA
South	Softwood	No	<25	3648	0.314	0.355	0.452	0.621
South	Softwood	No	25–50	2940	0.082	0.069	0.085	0.072
South	Softwood	No	50–100	2345	0.039	0.049	0.039	0.050
South	Softwood	No	≥100	673	0.021	0.050	0.020	0.051
South	Softwood	Yes	<25	464	0.340	0.407	0.487	0.694
South	Softwood	Yes	25–50	348	0.081	0.071	0.084	0.074
South	Softwood	Yes	50–100	299	0.038	0.039	0.038	0.041
South	Softwood	Yes	≥100	110	0.020	0.038	0.020	0.039
South	Hardwood	No	<25	6585	0.133	0.191	0.176	0.291
South	Hardwood	No	25–50	6180	0.040	0.044	0.041	0.045
South	Hardwood	No	50–100	8244	0.021	0.032	0.021	0.032
South	Hardwood	No	≥100	2697	0.014	0.032	0.014	0.032
South	Hardwood	Yes	<25	630	0.140	0.184	0.185	0.272
South	Hardwood	Yes	25–50	498	0.042	0.062	0.044	0.064
South	Hardwood	Yes	50–100	756	0.021	0.029	0.021	0.030
South	Hardwood	Yes	≥100	275	0.011	0.029	0.011	0.029
West	Softwood	No	<25	56	0.061	0.102	0.079	0.123
West	Softwood	No	25–50	45	0.027	0.048	0.028	0.049
West	Softwood	No	50–100	61	0.022	0.026	0.022	0.027
West	Softwood	No	≥100	80	0.014	0.019	0.014	0.019
West	Softwood	Yes	<25	0	*0.310*	*NA*	*0.443*	*NA*
West	Softwood	Yes	25–50	0	*0.072*	*NA*	*0.075*	*NA*
West	Softwood	Yes	50–100	0	*0.037*	*NA*	*0.037*	*NA*
West	Softwood	Yes	≥100	0	*0.020*	*NA*	*0.020*	*NA*
West	Hardwood	No	<25	33	0.037	0.055	0.043	0.061
West	Hardwood	No	25–50	26	0.023	0.026	0.025	0.028
West	Hardwood	No	50–100	45	0.026	0.041	0.027	0.043
West	Hardwood	No	≥100	38	0.019	0.025	0.020	0.027
West	Hardwood	Yes	<25	0	*0.137*	*NA*	*0.180*	*NA*
West	Hardwood	Yes	25–50	0	*0.041*	*NA*	*0.043*	*NA*
West	Hardwood	Yes	50–100	0	*0.021*	*NA*	*0.021*	*NA*
West	Hardwood	Yes	≥100	0	*0.011*	*NA*	*0.011*	*NA*

*Italics* imputed from other regions

#### Disturbance maps (2006–2010)

Sources of disturbance data used in this analysis are summarized in Table 3 and include spatially-explicit data on locations of fire, insect damage, wind damage, land use change, drought, and timberlands. The timberlands map was used to attribute net carbon gains occurring within vs. outside timberland areas. Because harvested wood may come from intermediate treatments (treatments not intended to cause regeneration), partial harvest or clearcutting forests, deforestation, and non-forest land trees, the area of clearcuts as observed within timberland areas through remote sensing imagery cannot represent all these wood sources [28]. Therefore for estimating C losses from timber harvest, we used data collected in the US based on mill surveys rather than remote sensing observations.

**Table 3:** Fourteen independent datasets were integrated and used to produce net carbon change estimates by disturbance type

Product	Source	Spatial coverage	Temporal coverage	Url
Tree cover Tree cover change	[8]	Complete CONUS	Tree cover: single snapshot in 2000 Loss: annual 2001–2010 Gain: 2000–2012	http://earthenginepartners.appspot.com/science-2013-global-forest/download_v1.1.html
Fire	Monitoring trends in burn severity	Complete CONUS	Annual 2006–2010	http://www.mtbs.gov/products.html
Wind	NOAA’s storm prediction center—tornado tracks	Complete CONUS	Annual 2006–2010	http://www.spc.noaa.gov/gis/svrgis/
Wind	NOAA’s storm prediction center—hurricane paths	Complete CONUS	Annual 2006–2010	http://nhc.noaa.gov/gis/
Insect	USFS aerial detection survey	Sub-set of CONUS	Annual 2006–2010	http://www.fs.fed.us/foresthealth/technology/adsm.shtml
Forest type	National land cover database—hardwood or softwood	Complete CONUS	Single snapshot in 2000	http://www.mrlc.gov/
Conversion	National land cover database	Complete CONUS	Snapshots in 2006 and 2011	http://www.mrlc.gov/
Drought	NDMC drought monitor	Complete CONUS	Weekly between 2006 and 2011	http://droughtmonitor.unl.edu/
Timberlands	Mark Nelson USFS for 2007 resources planning act	Complete CONUS	Snapshot in 2007	N/A
Biomass density Carbon stocks	Sassan Saatchi	Complete CONUS	Snapshot in 2005	http://dx.doi.org/10.3334/ORNLDAAC/1313)
Harvest	USFS timber products output	Combined county CONUS	Survey in 2007	http://www.fia.fs.fed.us/programfeatures/tpo/
FIA	USFS forest inventory and analysis program	Sites in CONUS	Between 1997 and 2013	http://www.fia.fs.fed.us/

#### Timber product output data (TPO 2007)

The volume of roundwood products, mill residues and logging residues reported in the TPO database (Table 3), separated by product class and detailed species group, were used to estimate C losses from wood harvest. The spatial resolution of the data was the “combined county”, which represented the minimum reportable scale from the timber product output (TPO; FIA Fiscal Year 2013 Business Report, [29]) data while retaining necessary confidentiality.

### Model assumptions

#### IPCC Tier 2 estimation

The terrestrial C cycle includes changes in C stocks due to both continuous processes (i.e., growth, decomposition) and discrete events (i.e., disturbances such as harvest, fire, insect outbreaks, land-use change). Continuous processes can affect C stocks in all areas every year, while discrete events (i.e., disturbances) cause emissions and redistribute C in specific areas in the year of the event. In accounting for net C change in this analysis, we use country-specific data (Tier 2) and apply the simplifying methodological assumption [15] that all post-disturbance emissions (after accounting for C storage in harvested wood products) occur as part of the disturbance event, i.e., in the year of disturbance, rather than modeling these emissions through time as in IPCC’s Tier 3 approach. The application of lower tier methods also assumes that the average transfer rate into dead organic matter (dead wood and litter) is equal to the average transfer out of dead organic matter, so that the net stock change in these pools is zero [15]. This assumption means that dead organic matter (dead wood and litter) C stocks need not be quantified for land areas that remain forested. The rationale for this approach is that dead organic matter stocks, particularly dead wood, are highly variable and site-specific, depending on forest type and age, disturbance history and management. Because the FIA data used in this analysis do not include measurements of soil C or dead C pools and no robust relationships currently exist that relate these pools to a more easily measured pool (such as the derivation of belowground biomass from aboveground biomass using root:shoot ratios), we excluded the soil C and dead C pools from our analysis. As a result, our estimate of net C change using the stock-difference approach is equal to the net change in C stocks in the aboveground and belowground live biomass pools only, with a fraction of the aboveground live biomass assumed to be transferred to the wood products pool, where a portion is permanently sequestered in long-lived products and the remainder emitted to the atmosphere (see below).

#### Disturbance attribution

Forest land was assumed to be disturbed if included in at least one of the disturbance maps (Table 3) during the 2006–2010 time period: (1) maximum burn severity score of at least two (low) over the 5 years of fire data; (2) insect damage of at least three trees per acre over the 5 year study period; (3) within a path of a tornado or a buffered region around the hurricane path where wind speeds typically exceeded 95 miles per hour (category 2 hurricane)^2^ between 2006 and 2010; (4) converted to agriculture, barren land or settlement in the NLCD layer between 2006 and 2011 (considered as deforestation events); or (5) had an average drought intensity score of more than two in the NDMC Drought Monitor map between the years of measurement. For fire and insect disturbance, three levels of disturbance intensity were assigned based on burn severity score (from the MTBS dataset) or insect damage per acre (from the Aerial Detection Survey), respectively. Two levels of wind disturbance intensity were assigned and areas determined to have been converted to agriculture or settlement were assumed to experience one uniform intensity of disturbance. All other forest land was assumed to be undisturbed between 2006 and 2010. In areas where multiple types of disturbance were identified within a 1 ha forest land pixel, we assumed only one disturbance type was driving the C loss. Disturbance type priority was set based on the intensity of the disturbance and level of confidence in the data sets. In general, more intense disturbances and higher quality products took priority over less intense disturbances and those products assessed as having more uncertainty. The disturbance location and intensity products were assumed to be in the following quality order, from least to most inherent uncertainty: conversion, fire, wind, insect damage. For instance, a pixel identified as experiencing an intense fire disturbance and a low intensity insect disturbance was assigned the high intensity fire disturbance as the single disturbance driving loss. This assumption simplified the processing but added additional uncertainty to the estimates. The assigned disturbance type priority varied across multiple iterations of our uncertainty analysis. It was not possible to attribute harvest disturbance to specific pixels, therefore C losses from harvest were estimated at the county scale using TPO data.

### Estimation of net carbon change

#### Net carbon change from fire, wind, insect damage, land use change, and drought

If a hectare of forest land in the US was categorized as disturbed between 2006 and 2010 based on the disturbance maps, then the intensity and type of disturbance was identified. The pixel was then linked to an annualized percent net change in C stock estimate, based on its identified category in the FIA-based lookup tables. These annualized percent change values were multiplied by the initial base C stock in 2005 in each pool (aboveground biomass, belowground biomass) and multiplied by 5 years to estimate total net change in C within the pixel between 2006 and 2010.

#### Net carbon change from harvest

Annual C losses associated with harvest activities were estimated using mill surveys compiled into the USDA TPO database for the year 2007. Due to the periodic nature of the TPO report for 2007 data, harvest emission estimates were assumed to be representative for all 5 years included in our analysis (2006–2010). Volumes of roundwood products, mill residue and logging residues were converted to biomass using oven-dry wood densities [30]. The fraction of C in primary wood products remaining in end uses or in landfills after 100 years per product class^3^ was assumed to be permanently sequestered, and was estimated from values published in Smith et al. [31]. Fuelwood, posts/poles/pilings and miscellaneous product classes were assumed to be fully emitted. Emissions from mill residues were considered equal to the summed mill residues from fuel by-products, miscellaneous by-products and unused mill residues, plus emissions from fiber by-products. All fiber by-products were assumed to form pulp and to follow the emissions assumptions of pulp products. All logging residues were assumed to be emitted. Timberlands were delineated based on the boundaries of the US timberlands map (Table 3), and annual net C gains within timberlands were estimated following the look-up tables for growth in undisturbed forests as described below.

#### Net carbon change from forest growth/regrowth

Forest land in the US that did not experience deforestation through land use conversion or significant damage by wind, insect, fire, or drought over the analysis period, as well as new forest land (i.e., afforestation/reforestation), were linked to values of annual net change in C stock, based on the area’s identified category in the lookup tables derived from FIA measurement data. These annualized percent change values were multiplied by the initial C stock in 2005 in each pool (aboveground biomass, belowground biomass) and multiplied by 5 years to estimate total net change in C within each 1-ha pixel between 2006 and 2010.

#### Total annual net carbon change

The FIA-based estimated net change in C represents the sum of net C losses (caused by disturbances) and net C gains (caused by forest growth) that occurred between FIA measurement dates at the site. Similarly, our estimate of net C change (ΔC_net_) during the 5-year period at the combined county scale was calculated as: $$\begin{aligned}\Delta {\text{C}}_{\text{net}} &= \Delta {\text{C}}_{\text{undist}} + \Delta {\text{C}}_{{{\text{A}}/{\text{R}}}} + \Delta {\text{C}}_{\text{conversion}} \\ & \quad + \Delta {\text{C}}_{\text{timberlands}} + \Delta {\text{C}}_{\text{insect}} + \Delta {\text{C}}_{\text{fire}} \\ & \quad + \Delta {\text{C}}_{\text{wind}} + \Delta {\text{C}}_{\text{drought}} \end{aligned}$$

where ΔC_undist_ is the net C change in forest land outside of timberlands that did not experience land use conversion or significant damage by wind, insects, fire or drought. ΔC_A/R_ is the net C change in new forest land. ΔC_conversion_, ΔC_wind_ ΔC_insect_, and ΔC_fire_ represent the net C change in forestland that was converted or significantly disturbed by conversion, wind, insects, and fire, respectively. ΔC_drought_ is the net C reduction in sequestration in forest land experiencing drought from what was expected during non-drought periods. ΔC_timberlands_ is the net C change on timberlands (as delineated by the timberlands map), calculated as the sum of net C gains (as estimated from FIA lookup tables) and C losses (as estimated from the TPO data, accounting for the fraction of harvested C stored permanently in the long-lived product pool). By convention, C losses are represented as positive values and C gains as negative values. Consequently, various forms of disturbance result in a weaker (i.e., less negative) overall sink than would occur otherwise in the absence of disturbance.

### Uncertainty analysis

We estimated statistical bounds for the estimates of net C change by conducting a Monte Carlo uncertainty analysis [32]. The four sources of uncertainty included in the simulation were associated with the forest biomass density maps, the stock-change lookup tables derived from FIA data, each of the disturbance maps, and the TPO data. The simulation was conducted at the combined county scale. Uncertainty in the biomass density maps was derived from a secondary simulation in which the input datasets were resampled to generate 100 replicate training datasets, or realizations, that had the same qualities of the original training dataset, but different random error. A new MaxEnt model was fit to each of these 100 replicated datasets and used to create 100 full resolution biomass maps. Uncertainty in the FIA-based ΔC values were calculated using the variance in the look-up tables:

$$uncertainty\% = \frac{{\frac{\sigma }{\sqrt n }*1.96}}{\mu }*100$$

Uncertainty in the area affected by disturbance was estimated to be 30%, with an estimated 5% bias in under reported area. We conducted the simulation using three separate rule sets for selecting a disturbance type for pixels identified as experiencing multiple disturbances during the 5-year study period. Uncertainty in the TPO data at the combined county scale was also assumed to be 30%.

We ran 10,000 Monte Carlo simulations with stochastic elements in place for the four uncertainty components. We assumed that 80% of the randomly generated error was random and 20% of the error was systematic within the simulation. To implement this assumption, we estimated the error associated with each component twice—once at the simulation iteration level and again for each individual combined county. The iteration level uncertainty was multiplied by 0.2 before it was added to the original combined county estimate, while the combined county level stochastic element was multiplied by 0.8 before it was added. In this way, we accounted for both random error as well as systematic error in our estimates.

This uncertainty analysis was intended to provide context to the estimates and assist in the process of identifying methods and data in need of refinement or replacement. The uncertainty analysis is not exhaustive, in the sense that additional sources of uncertainty exist that are not accounted for in the analysis presented here. These additional sources include but are not limited to (a) potential temporal mismatch between the biomass data providing initial carbon stocks in 2005 and the activity data beginning in 2006 and (b) uncertainty in the equations and factors used in the FIA to convert tree measurements to estimates of wood volume and carbon stocks. Given these additional sources of uncertainty, the uncertainty bounds presented here are almost certainly an underestimate of the actual uncertainty.

## Results

**Fig. 2:**
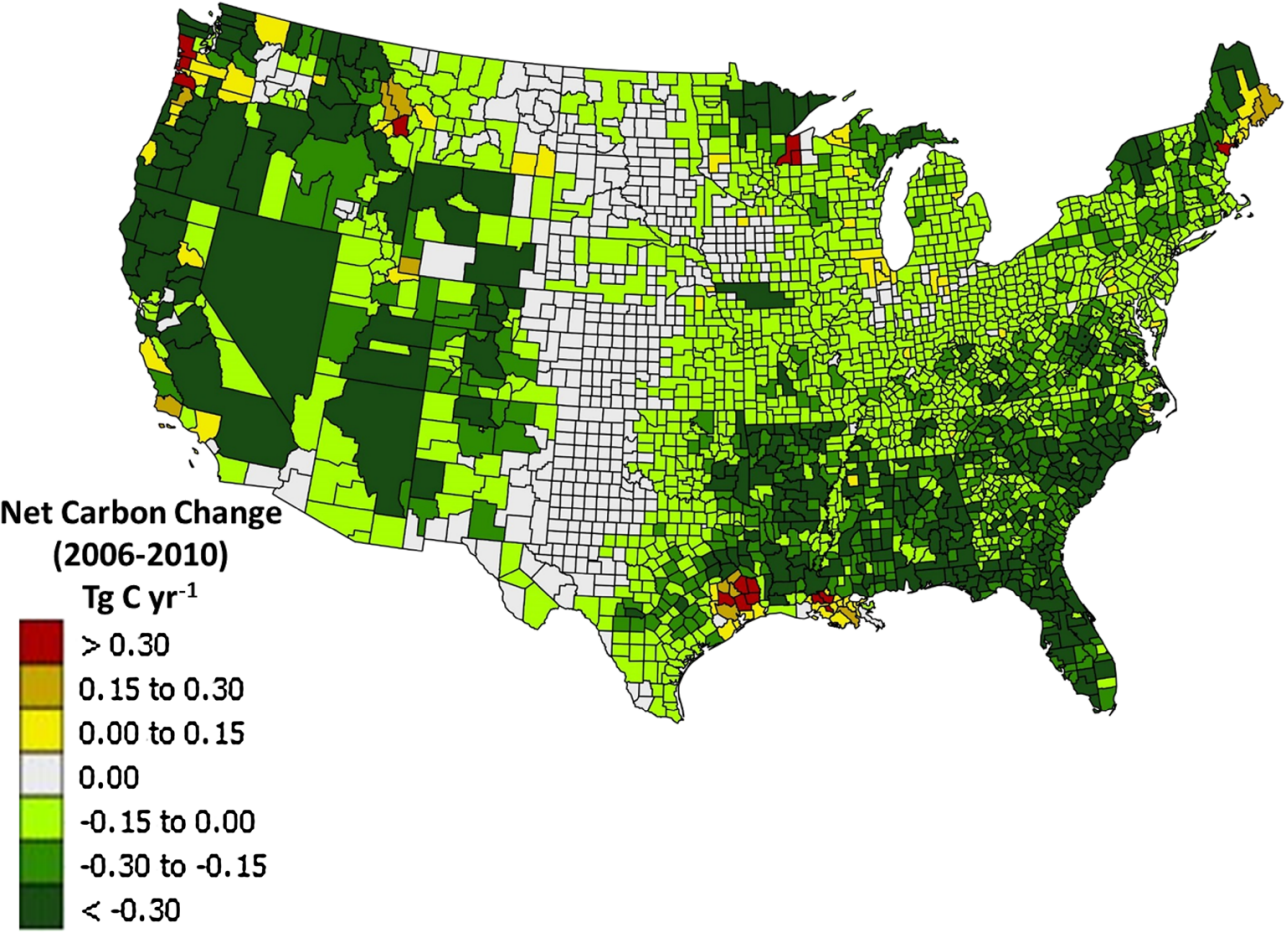
Average annual net carbon change (Tg C year^−1^) at the combined county scale across the CONUS. Most combined counties (91%) are net C sinks while areas with extensive forest disturbance can be net C sources to the atmosphere

Forest land in the conterminous US, as defined here totaling 221 million ha in 2005, sequestered −460 ± 48 Tg C year^−1^ between 2006 and 2010, while average C losses from forest disturbances were 191 ± 10 Tg C year^−1^. Combining estimates of net C gains and net C losses results in net C change of −269 ± 49 Tg C year^−1^ (Fig. 2). These results are broadly consistent with estimates reported in the US. GHG inventory for forests in 2010 (−293 Tg C year^−1^, [33]) but we estimate a larger net sink than reported in Zheng et al. [28] (−181 Tg C year^−1^), although the spatial and temporal domains varied across these analyses, as did the C pools included.

New forests, averaging 0.4 million ha per year, sequestered −8 ± 1 Tg C year^−1^, while deforestation, averaging 0.1 million ha per year, resulted in C losses of 6 ± 1 Tg C year^−1^. Forest land remaining forest land lost 184 ± 10 Tg C year^−1^ to disturbance (13% from natural disturbance, 87% from harvest); these were compensated by net carbon gains of 452 ± 48 Tg C year^−1^, 75% of which occurred within timberland areas (Table 4). C losses from natural and human induced disturbances reduced the potential net C sink in US forests by 42% compared to the potential sink estimated without disturbance effects included, an estimate that is similar to other studies [28, 34].

**Table 4:** Average annual net C change (Tg C year^−1^) across US forests between 2006 and 2010, disaggregated into categories of non-forest land to forest land, forest land to non-forest land, and forest land remaining forest land

Category	Area (Mha year^−1^)	Net C gain (Tg C year^−1^)	Net C loss (Tg C year^−1^)
Non-forest land to forest land	0.4	−8 ± 1	
Forest land to non-forest land	0.1		6 ± 1
Forest land remaining forest land	221.1	−452 ± 47	185 ± 10
Insect damage	0.9		9 ± 1
Forest fire	0.6		7 ± 1
Wind damage	0.6		5 ± 1
Drought	0.8		1 ± 0
Timberlands	152.0	−342 ± 42	162 ± 10
Undisturbed forest	54.9	−109 ± 19	
Total	221.6	−460 ± 48	191 ± 10
Net C change			−269 ± 49

Results are further disaggregated by disturbance type within the forest land remaining forest land category

Regional variation in net C change across the nation was substantial. The South sequestered more C in growing forests (−271 ± 28 Tg C year^−1^) than the North (−97 ± 10 Tg C year^−1^) or the West (−92 ± 11 Tg C year^−1^), while at the same time losing more C to the atmosphere from disturbances (105 ± 6 Tg C year^−1^) than the other regions (41 ± 2 Tg C year^−1^ for the North and 44 ± 3 Tg C year^−1^ for the West). Forest C change in the South was substantial, in terms of both C losses and gains, because this region is home to a majority of the wood harvest occurring in the US (60% of all C loss from harvest occurred in the South), and is therefore also home to the largest area of regenerating forests that are sequestering C at high rates. At the state level, the highest C losses occurred in the forests of Georgia, Alabama, Washington, Mississippi, Louisiana, and Oregon, with each of these states losing more than 11 Tg C year^−1^ (Table 5). Georgia, Florida, Alabama, Mississippi, and North Carolina gained the most forest C in the time period, with each sequestering at least 24 Tg C year^−1^. C gains exceeded C losses in all states. Forests in approximately 6% of combined counties were a net source of C to the atmosphere (Fig. 2).

**Table 5:** State level estimates of forest area in 2005 (millions of ha), net C gains, net C losses, and net C change (Tg C year^−1^) together with the percent of C loss attributable to harvest, drought, fire, wind, insect infestation, and land use conversion within the state

State	Forest area	C gain	C loss	Net C change	Fire (%)	Insect (%)	Wind (%)	Conversion (%)	Drought (%)	Harvest (%)
Alabama	8.5	−27.3	12.5	−14.9	0	1	0	1	0	97
Arizona	2.0	−2.4	0.4	−1.9	22	0	1	0	0	77
Arkansas	7.4	−22.6	8.6	−14.0	1	2	0	2	0	95
California	9.3	−16.8	9.4	−7.4	32	0	7	1	0	60
Colorado	5.1	−6.7	0.3	−6.3	8	0	0	1	0	92
Connecticut	0.9	−1.2	0.2	−1.0	0	0	1	31	0	68
Delaware	0.2	−0.2	0.1	−0.1	0	0	0	4	0	95
District of Columbia	<0.1	0.0	0.0	0.0	0	0	0	100	0	0
Florida	6.4	−28.5	6.3	−22.2	3	0	0	3	0	94
Georgia	9.4	−33.2	14.4	−18.8	1	1	0	2	0	96
Idaho	7.1	−10.2	4.9	−5.3	29	0	23	0	0	48
Illinois	2.3	−2.8	1.1	−1.7	0	0	0	3	0	97
Indiana	2.3	−2.8	1.7	−1.1	0	0	3	1	0	95
Iowa	1.2	−1.5	0.4	−1.1	0	1	0	3	0	96
Kansas	0.9	−1.1	0.2	−0.9	0	1	0	3	0	95
Kentucky	5.7	−11.5	3.3	−8.2	1	0	0	6	0	93
Louisiana	5.4	−18.0	11.1	−6.9	0	19	0	1	0	79
Maine	6.8	−7.7	6.7	−0.9	0	0	15	1	0	84
Maryland	1.2	−1.5	0.8	−0.8	0	0	6	7	0	86
Massachusetts	1.5	−1.9	0.6	−1.3	0	0	4	18	0	78
Michigan	8.5	−10.3	4.3	−6.0	0	0	1	1	11	87
Minnesota	7.7	−9.5	3.2	−6.3	1	0	3	1	0	96
Mississippi	7.0	−24.3	11.6	−12.7	0	2	0	2	0	96
Missouri	7.1	−8.7	2.7	−6.0	1	2	0	4	0	93
Montana	7.3	−8.6	5.0	−3.5	14	0	49	0	0	37
Nebraska	0.3	−0.4	0.1	−0.2	2	1	0	0	0	97
Nevada	0.7	−0.8	0.1	−0.7	15	0	0	0	0	84
New Hampshire	2.1	−2.6	0.8	−1.8	0	2	4	6	0	88
New Jersey	1.0	−1.3	0.5	−0.8	2	0	40	14	0	43
New Mexico	2.6	−3.2	0.3	−2.8	33	0	16	0	0	51
New York	8.3	−10.7	3.1	−7.6	0	0	5	4	0	91
North Carolina	7.6	−23.7	9.6	−14.1	0	0	0	1	2	95
North Dakota	0.2	−0.3	0.0	−0.3	0	1	0	2	0	96
Ohio	3.6	−4.4	1.2	−3.2	0	0	7	7	0	86
Oklahoma	3.6	−9.0	1.6	−7.3	2	2	0	3	0	94
Oregon	9.2	−20.6	11.1	−9.6	4	0	2	6	0	88
Pennsylvania	7.6	−9.8	4.0	−5.8	0	0	13	3	0	84
Rhode Island	0.2	−0.2	0.1	−0.2	0	0	3	11	0	85
South Carolina	4.8	−18.4	6.5	−11.9	1	1	0	2	0	97
South Dakota	0.5	−0.6	0.2	−0.3	2	0	0	0	0	98
Tennessee	6.2	−14.2	4.0	−10.1	0	1	0	3	0	95
Texas	7.9	−23.3	9.8	−13.6	1	23	0	2	0	74
Utah	2.2	−2.2	0.3	−1.8	24	0	38	0	0	38
Vermont	2.0	−2.5	0.6	−1.9	0	0	2	1	0	96
Virginia	6.7	−16.5	6.1	−10.4	1	0	0	2	0	97
Washington	7.9	−17.3	11.7	−5.6	3	0	8	19	0	70
West Virginia	5.3	−6.9	2.5	−4.4	0	0	1	6	0	93
Wisconsin	7.2	−8.4	6.3	−2.0	0	1	23	0	5	70
Wyoming	2.7	−3.3	0.8	−2.5	21	0	25	0	0	54
Total	221.5	−459.5	191.1	−268.4	4	3	5	3	1	85

**Fig. 3:**
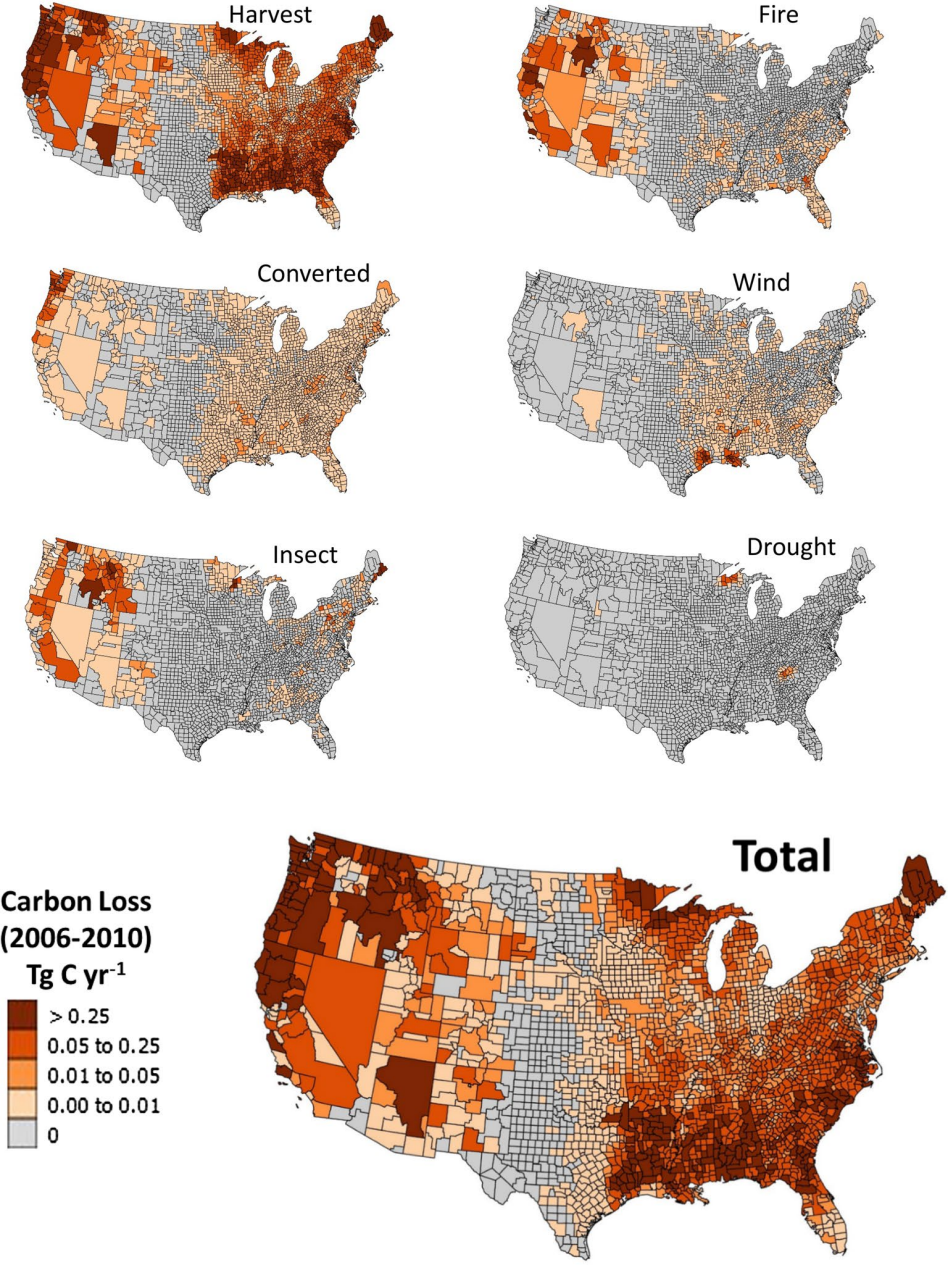
Average annual net carbon loss (Tg C year^−1^) attributed to the most likely disturbance type and estimated at the combined county scale for harvest, fire, land use conversion, wind, insect, and drought. Combining these six sources results in estimates of total annual net C loss from disturbance occurring between 2006 and 2010

**Fig. 4:**
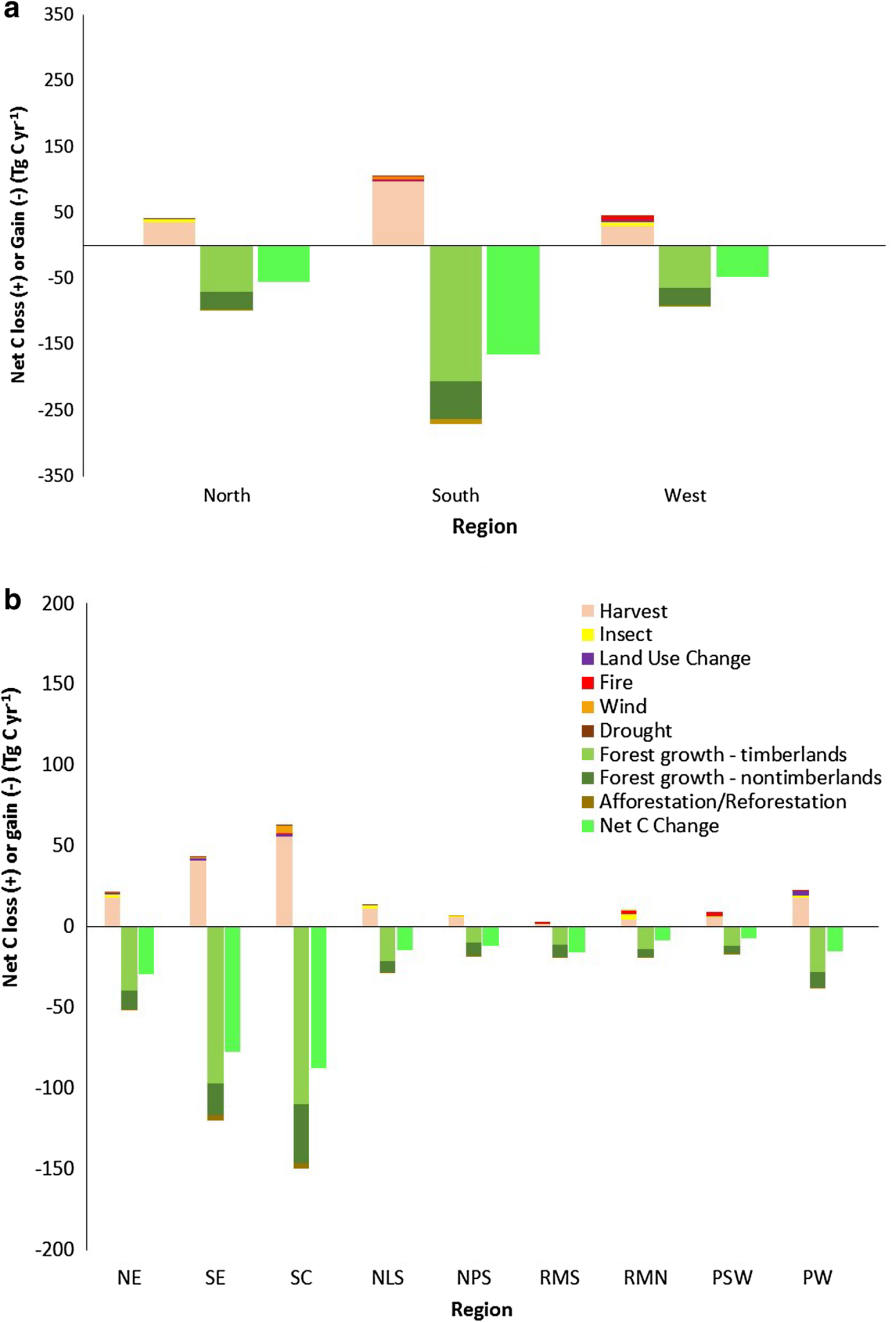
Average annual net carbon change by disturbance type in **a** the North (79 million ha of forest), South (87 million ha), and West (56 million ha) regions and **b** by FIA region: northeast (NE; 41 million ha), southeast (SE; 35 million ha), southcentral (SC; 52 million ha), northern lake states (NLS; 23 million ha), northern plains states (NPS; 15 million ha), pacific west (PW; 17 million ha), rocky mountain northern (RMN; 14 million ha), rocky mountain southern (RMS; 15 million ha), and the pacific southwest (PSW; 9 million ha)

We estimated net C losses from six separate disturbance processes: fire, insect infestation, wind, timber harvest, land use conversion, and drought (Fig. 3). C losses from harvest (162 ± 9.9 Tg C year^−1^) were more than five times higher than losses from all other processes combined (30 ± 2.6 Tg C year^−1^). Fire (7 ± 1.0 Tg C year^−1^), wind (5 ± 0.7 Tg C year^−1^), insect infestation (10 ± 1.3 Tg C year^−1^), and deforestation (6 ± 0.7 Tg C year^−1^) each contributed a similar magnitude of C losses across the CONUS, while drought accounted for about 1 ± 0.2 Tg C year^−1^. Individual disturbances had spatially distinct distributions (Fig. 4a). On average, drought affected areas had C sequestration rates 20% lower than drought-free areas.

C losses in the South were highest (105 ± 6 Tg C year^−1^) with the highest fractional contributions from harvest (92%) and wind (5%), with a particularly high concentration of loss coming from the South Central region (including the states of Texas, Oklahoma, Mississippi, Louisiana, Kentucky, Tennessee, Alabama, and Arkansas; Fig. 4b). The West had the second highest C loss (44 ± 3 Tg C year^−1^) with significant contributions from harvest (66%), fire (15%), and insects (13%). The North had the lowest C loss (41 ± 2 Tg C year^−1^) with most significant proportional contributions coming from harvest (86%), insect damage (9%), and conversion (3%).

**Fig. 5:**
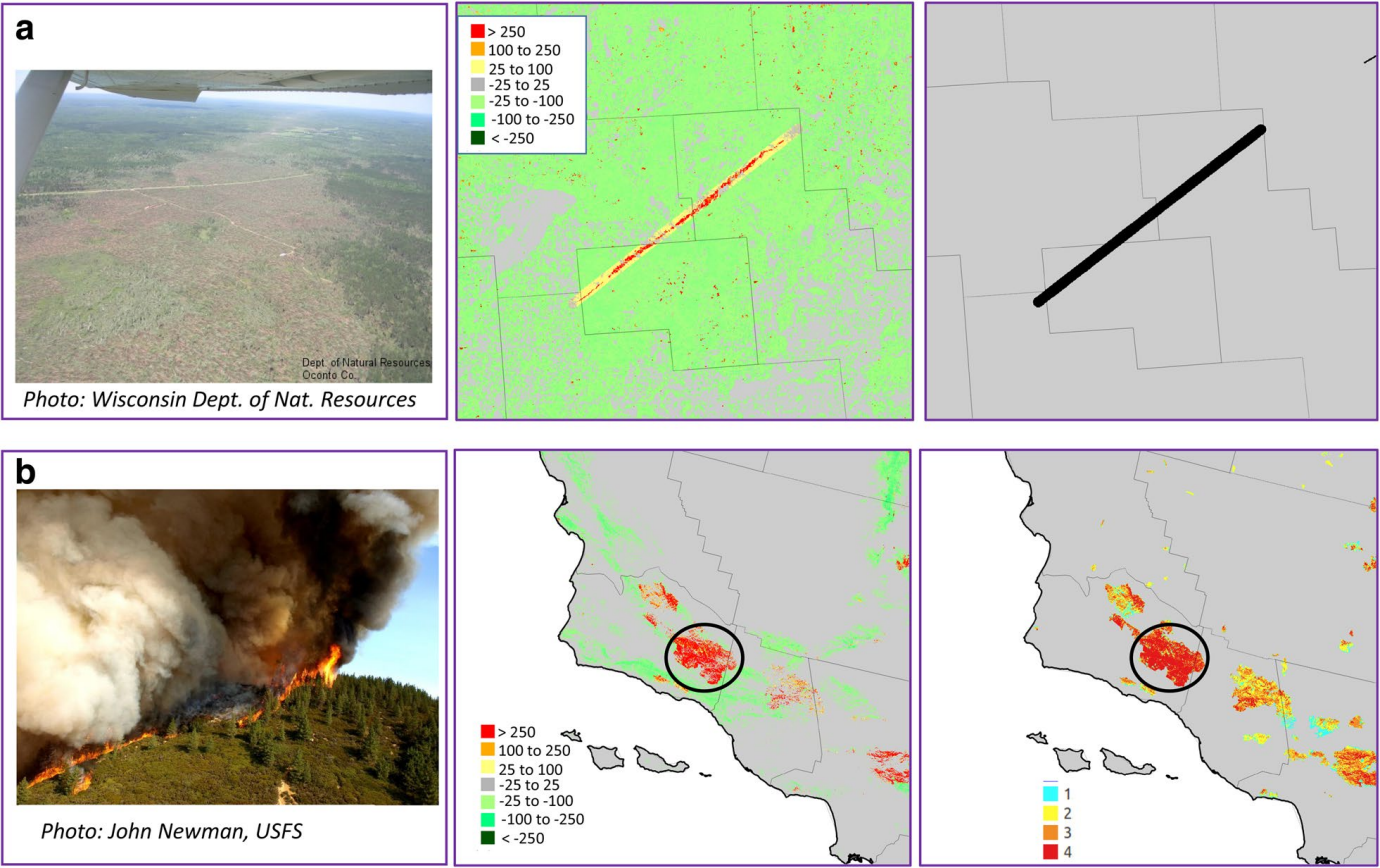
The forest carbon accounting framework implemented here can be useful in assessing carbon impacts of localized disturbances. **a** 2007 tornado in Lakewood, Wisconsin. The tornado track from NOAA (*right*) resulted in extensive impacts to the forest, which is evident in an aerial photo (*left*) and in the resulting estimate of net carbon change (center, in units of Mg C ha^−1^). **b** 2007 wild fire in southern California’s Santa Barbara County, termed the “Zaca” fire. A photo of the blaze (*left*) highlights the fire intensity, which is mirrored in the burn severity map (*right*, MTBS) and the resulting net carbon change estimate (*center*, in units of Mg C ha^−1^)

Our results can also be used to estimate net C impacts of localized disturbances at finer spatial scales. A tornado struck Lakewood, Wisconsin on 7 June 2007 and caused severe forest damage, resulting in net C loss of more than 0.3 Tg C across a 13,000 ha swath (Fig. 5a). The wild fire in southern California’s Santa Barbara County, termed the “Zaca” fire, started on 4 July 2007 and caused extensive damage to more than 97,000 ha of forest in the Los Padres National Forest, resulting in net C loss of more than 4 Tg C (Fig. 4b).

**Fig. 6:**
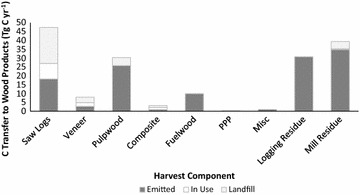
Fate of C harvested from US forestlands in the year 2007, with some stored in use and landfills and the rest emitted within 100 years. *PPP* posts, poles and pilings

The highest fractional contribution of C loss in all states was from harvest (Table 4), and 64% of these losses were from logging residues [both above- (19%) and belowground (23%)] and mill residues (22%). Across all wood product classes, the production of pulpwood resulted in the highest forest C losses (26 Tg C year^−1^), followed by saw logs (18 Tg C year^−1^), although a high proportion of C in saw logs is in use or in landfills, both which are considered to be long-term C storage (Fig. 6).

## Discussion

### Comparison with other studies

We estimate that Hurricanes Gustav and Ike in 2008, the only two hurricanes above category 2 to make landfall during the study period, damaged forests in Texas and Louisiana and led to net C change of more than 22 ± 2 Tg C (or 4 ± 0.5 Tg C year^−1^ on average over the 5 year period). Other studies report average annual C loss in US forests due to hurricane damage in the 20th century of 14 Tg C year^−1^ [35]. Zhou et al. [36] estimate total C emissions from wood harvest in 35 eastern US states as 168 Tg C year^−1^ between 2002 and 2010, while our estimate for the same geographic extent is 132 ± 8 Tg C year^−1^ between 2006 and 2010. Other national scale estimates of emissions from wood harvest are lower, such as that of Williams et al. [37] (107 Tg year^−1^ in 2005) and Powell et al. [34] (74 Tg C year^−1^ between 1986 and 2004). Hicke and Zeppel [38] estimated that bark beetles and fire together resulted in gross emissions of 32 Tg C year^−1^ in the western US between 1997 and 2010. We estimate that insects and fire resulted in net C change of 17 ± 2 Tg C year^−1^ between 2006 and 2010. We conclude that, given the different spatial extents, time periods and C pools included, results from our analysis that cover all disturbance types are broadly consistent with these and other more specialized studies (see Williams et al. [39] for a comprehensive review).

### Priorities for improved forest carbon change estimates

Results generated from this analysis are dependent on the algorithm that assigns each hectare of forest land to a category that is then associated with a C stock change value. By including spatial data sets of carbon stocks and disturbance from remote sensing observations, the methodology avoids making gross assumptions on the regional distribution of carbon stocks and disturbance, thus improving estimates of C loss. The strength of this approach is estimated in the uncertainty analysis. Our framework is therefore completely dependent on the underlying data sources and, as the data improve, so will the estimates. Although the US is among the world’s leaders in technology and open data, where high quality geospatial datasets are publicly available and inventory programs are maintained by various federal and state agencies, opportunities for improvement remain.

#### Priorities for FIA data collection

All forest inventory data used to estimate changes in the above- and belowground C stocks in this analysis come from FIA plots measured more than once. However, many more FIA plots have been re-measured in the North and South regions of the US than in the West. The limited number of re-measured FIA plots in the West resulted in higher uncertainties in net C stock change estimates and, in some disturbance categories, required the imputation of estimates obtained from other regions (Tables 1, 2). As the FIA program continues national implementation of an annual inventory (including re-measurement), the FIA data used in this analysis can be revised accordingly so that the sample size of plots per disturbance type increases and uncertainties decrease. Until the early 2000s, the FIA program measured only live tree attributes (e.g., tree diameter) allowing for the estimation of aboveground C and modelling of the other pools based on regions, live tree, and site characteristics (although the dead wood pool was measured in some states). Therefore, we estimated changes in the aboveground C pool using measured data while we relied on models to estimate belowground C. The FIA program is in the process of replacing model predictions of C in the dead wood, litter, and soil organic C pools with estimates obtained from measurements of these pools on a subset of FIA plots [40]. These pools, excluded from the current analysis, can be included in our framework as new data are collected.

#### Priorities for non-forest lands

Our analysis focused on forest areas defined in part by the NLCD data that is based on the interpretation of Landsat imagery. Comparison of our 1-ha map of carbon density of forestlands based on NLCD with high resolution lidar data over the state of Maryland has shown a significant underestimation of carbon stocks in highly fragmented and mixed urban and forest landscapes [41]. These small scale forests cover substantial areas of densely populated and fragmented landscapes of the eastern United States and appear to be highly dynamic. There is information on the disturbance and recovery of these forests over the time frame of our study, but our analysis has ignored carbon sources and sinks from these lands. By improving the carbon inventory and satellite observations to capture small scale changes, the uncertainty of carbon fluxes, particularly over the Eastern states, may be reduced. In the future (post-2020), planned satellite observations of the aboveground structure of forests by GEDI and NISAR from the National Aeronautics and Space Administration (NASA) and BIOMASS from the European Space Agency should improve the annual inventory of forest C change, as should the planned collection of FIA plot data in urban and woodland areas.

#### Priorities for UNFCCC reporting

Although the US has data on the magnitude of area change across land use categories, it does not have reliable and comprehensive estimates of C stocks across the entire reporting time series (e.g., 1990–2014 for the most recent UNFCCC submission) and full matrix of land use and land-use change categories to report these changes separately. For this reason, in its GHG inventory submission the US has historically deviated from IPCC guidance by reporting together C stock changes from afforestation and forest management as “forest land remaining forest land”, while emissions associated with a land use conversion from forest land to a non-forest land use are reported in the non-forest land use category (per IPCC guidance). For the first time in its 2016 submission [16, 17], the US delineated net C stock changes from afforestation separately from forest land remaining forest land. An additional data need is refined C stock monitoring on non-forest lands and better coordination among land use categories to ensure complete accounting and avoidance of double counting. Our spatially resolved analysis approach allowed us to disaggregate net C change into subcategories of non-forest land to forest land (−8 ± 1 Tg C year^−1^), forest land to non-forest land (6 ± 1 Tg C year^−1^), and forest land remaining forest land (−267 Tg C year^−1^). While the sole focus on net processes within the forest land use category in this study does not fully solve complete C accounting issues across all land uses, the methods used in this research are an incremental improvement toward resolving components of net C change within the forest land category, and these results can help inform and refine US reporting in the future.

#### Priorities for improving disturbance attribution

Insect and disease aerial detection surveys (ADS) are conducted annually using a variety of light aircraft by the USDA Forest Service in collaboration with other state and federal cooperators. Overview surveys map the current year’s forest impact, and some regions have been conducting ADS for more than 60 years while others have become more active only within the last decade. Therefore, annual maps of insect damage with full coverage of all US forestlands are not available, but areas most likely to be affected by insect damage are surveyed more frequently. We accounted for the lack of continuous data coverage in our uncertainty analysis by assuming a 5% bias in underreported area. The Monitoring Trends in Burn Severity (MTBS) dataset, sponsored by the Wildland Fire Leadership Council, consistently maps the burn severity and perimeters across all lands of the US since 1984. Although 30 m resolution imagery is used for analysis, the minimum mapping unit for delineating fire perimeters is greater than 1000 acres (404 ha) in the West and 500 acres (202 ha) in the East. Therefore, burned forest areas smaller than these patch sizes were excluded from our analysis.

#### Priorities for wood harvest data collection

**Fig. 7:**
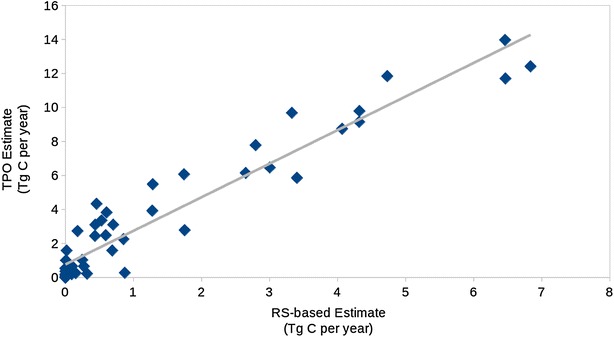
Relation between C losses from harvest as estimated from timber product output (TPO) data and from an independent remote sensing-based estimate. TPO = 1.98 × RS + 767,777; R^2^ = 0.91). Data points represent results aggregated to the state-level

Information on the primary anthropogenic source of C loss in US forests—wood harvest—is available only at the level of combined counties. TPO data allow for the estimation of C losses from the extraction of wood products that are not readily detected by remote sensing observations, including the most recent Landsat based tree cover loss data from Hansen et al. [8]. We examined the relationship between TPO estimated C losses and a remote sensing-based estimate of C losses from forest disturbance that could not be readily linked to another disturbance type (i.e. wind, insect, fire, or conversion). For this comparative analysis, we assumed all tree cover loss pixels in Hansen et al. [8] data that could not be linked to another disturbance type were harvested, and subsequent C loss was estimated via our FIA look-up table approach. When aggregated to the state level, these two independent estimates of C loss associated with harvest were highly correlated (Fig. 7), and the remote sensing-based estimates of (net) C loss from harvest were approximately half of the (gross) TPO-based estimates. This provides indications that: (1) Landsat-based remote sensing observations likely miss a significant proportion of harvest activity due to partial loss, rather than full loss, of tree canopy cover; and (2) the additional C loss not identified by the remote sensing approach is spatially proximate to larger scale C losses from harvest, at least at the state scale. Increased transparency on the spatial location, timing and type of harvesting occurring across the US would allow more explicit attribution of forest C fluxes to specific forest management activities.

### Managing US forests for climate change mitigation

Globally, the US ranks fourth in terms of forest area [42, 8]. Although large C losses occur from US forests as a result of an active wood products industry, particularly in the US South, 76% of the total US net carbon sink (342 Tg C year^−1^) occurred within timberland areas, more than half of which are privately owned [43]. The income received by landowners from Intensive forest management may reduce the likelihood of forest conversion to development, but in the absence of all disturbance effects, we estimate a potential C sink between 2006 and 2010 of −460 and −436 Tg C year^−1^ if only non-harvest disturbance effects (fire, drought, wind, insect damage, land-use conversion) are considered. The US has also committed to restoring 15 Mha of forest land [44], which could further increase the C sink capacity of US forests. This implies that the US C sink could be increased substantially if existing forest land were managed to achieve this goal.

In addition to sequestering and storing atmospheric carbon, US forests also generate wood products that support the energy, industry, transport and building sectors both domestically and internationally. Given that wood harvest represents the majority of C losses from US forests, increasing the US net forest C sink would require shifts in current forest management practices as well as more refined and disaggregated information to reduce the uncertainty of these estimates and resolve these with correct estimation of net C change. For example, national debate has grown over the production of wood pellets as a renewable energy source, particularly from the southeast US, with demand driven by European policies to reduce emissions of greenhouse gases and increase the use of renewable energy. Georgia, Florida, Alabama and Virginia currently account for nearly all US wood pellet exports [45]. Although wood pellets are claimed by the industry to be made from residues at lumber mills or logging sites, the industry’s growth could lead to a substantial increase in demand on Southern forests, potentially creating incentives to expand plantations. The potential of bioenergy to reduce greenhouse gas emissions inherently depends on the source of the biomass and its net land use effects; bioenergy reduces greenhouse gas emissions only if the growth and harvesting of the biomass used for energy sequesters carbon above and beyond what would be sequestered anyway [46]. This additional carbon must result from land management changes that increase tree C uptake or from the use of biomass that would otherwise decompose rapidly.

New global emphasis on climate change mitigation as one of the many benefits that forests provide gives US decision makers the opportunity to re-evaluate national and state policy agendas to consider not only the production of merchantable wood volume and biomass for bioenergy, but also enhanced C sequestration and storage for climate change mitigation. As recognized in the 2014 Farm Bill [47], there is a growing need to both reduce the uncertainty associated with estimating forest biomass and the associated monitoring of C dynamics across US forests. As it currently stands, the statistical power of detecting changes in forest C stocks exists only at large regional scales [48], disallowing the detection of C change at policy-relevant scales such as encountered in the pellet industry. Continued research to both downscale forest C inventories and correctly attribute C change to natural and anthropogenic disturbance events is needed to empower forest management policy decisions.

## Conclusions

Achieving a global, economy-wide “balance between anthropogenic emissions by sources and removals by sinks” [1] will require both more emission reductions and more C sequestration from the forest sector. Results from this analysis indicate the location and estimated magnitude of C losses from different disturbances in absolute and relative terms, and can be used to track more explicitly which losses result from natural or anthropogenic disturbances. Our national net C change estimate of −269 ± 49 Tg C year^−1^ is within the range of previously reported estimates, and provides spatially explicit estimates and attribution of changes to different types of disturbances. Data are synthesized from various US agencies into a common framework, which could improve inter-agency dialogue to ensure complete accounting and to avoid double counting within and between land use categories. This work may also improve collaboration that drives a more efficient and participatory process for allocating resources towards activities that meet common goals, including an increased focus on climate change mitigation. The methodological framework and accompanying results allow US policymakers and negotiators to better understand the causes of forest C change more completely so that they can participate more effectively in domestic policy discussions about forest management and monitoring as well as in international negotiations. Integration of results from this and other studies should further enable the development of future US GHG inventories that include disturbance attribution and full land use change accounting in expectation of post-2020 commitment requirements.

## Footnotes

1 Within each 1 ha pixel, the wet woodland class was included as forest but was not used to determine whether the pixel was hard- or softwood. Hard- or softwood was determined based on the plurality of NLCD hard- or softwood 30 m pixels within the hectare, ignoring the sub-fraction of wet woodlands and selecting softwood when hard- and softwood fractions were equal.

2 This wind speed threshold was selected based on the Saffir Simpson Hurricane Wind Scale, which indicates that trees start to be uprooted and fall at category 2 sustained wind speeds between 96 and 110 mph. The hurricane tracks were buffered to a symmetrical width of 100 km.

3 The TPO and Smith et al. [31] product classes were mapped to one another as follows: Sawlog = softwood/hardwood lumber (depending on species); veneer = softwood plywood; pulp = paper; composite = oriented strandboard.
